# The dual-burden of professional and academic stress: a cross-sectional mapping of mental health status and coping efficacy among postgraduate students in Nairobi, Kenya

**DOI:** 10.3389/fpsyt.2026.1820905

**Published:** 2026-05-12

**Authors:** Joseph O. Onyango, Laura B. Angwenyi

**Affiliations:** Institute of Healthcare Management, Strathmore University, Nairobi, Kenya

**Keywords:** academic stress, COPE inventory, coping strategies, DASS-21, higher education, Kenya, mental health status, Nairobi

## Abstract

**Background:**

Postgraduate students face a dual burden of intense academic rigour and professional responsibilities, a dynamic particularly pronounced in growing economic hubs such as Nairobi, Kenya. While global literature highlights rising psychological distress in higher education, there is limited evidence on how specific coping mechanisms mediate mental health outcomes among postgraduates at private African universities. Understanding these dynamics is critical for institutional psychosocial support systems.

**Objectives:**

This study aimed to assess the mental health status (depression, anxiety, and stress) of postgraduate students at Strathmore University. Grounded in Lazarus and Folkman’s Transactional Model of Stress and Coping Mechanisms.

**Methods:**

Adopting a quantitative cross-sectional descriptive design, data were collected from 242 actively enrolled postgraduate students using a random stratified sampling technique. Mental health status was measured using the Depression, Anxiety, and Stress Scale (DASS-21), and coping mechanisms were evaluated via the Coping Orientation to Problems Experienced (COPE) inventory. Data analysis utilised descriptive statistics, Spearman’s rank correlation, and multiple linear regression models.

**Findings:**

Results indicated significant psychological strain, with participants reporting difficulty winding down and high levels of anticipatory anxiety (DASS means ranged from 2.23/to 2.73 on a scale of 0-3. Regression analysis showed that coping strategies accounted for 40.2% of the variation in mental health. Notably, emotion-focused coping (β = 0.307, p <.001) and avoidant coping (β = 0.344, p <.001) had significant positive effects on mental well-being in this context. Conversely, problem-focused coping (β = -0.189, p = .002) had a significant negative effect, suggesting that direct action-oriented strategies may exacerbate distress for students facing stressors beyond their immediate control.

**Conclusion:**

The study finds that postgraduate mental health is profoundly influenced by the “plasticity” of coping strategies. While active problem-solving is traditionally encouraged, for the Kenyan postgraduate master’s student, adaptive emotion-focused and strategic avoidant strategies currently offer greater psychological relief. Universities must move from generic support to “fit-for-purpose” interventions that destigmatise mental health services and promote adaptive emotional regulation to ensure academic and professional success.

## Introduction

Optimal mental health is a precondition for health, happiness and satisfaction in life. It influences an individual’s capacity to adapt to difficult situations and to enjoy gratifying moments. While physical health has historically been the main focus for interventions, in recent years, mental health has gained recognition as a critical component of global development and human well-being, prompting growing international commitment to address it systematically and equitably. The World Health Organisation explains mental health as “a situation where a person is able to contribute and give back to their community by working productively, and realising their potential while coping with the normal pressures of life (1). There are three major components of mental health: cognitive health, which comprises a person’s thoughts; emotional health, which includes attitudes and feelings; and behavioural health, which examines an individual’s interactions with their environment and the strength of their relationships (2).

Mental illness is recognised as a major contributor to the global disease burden (3), with the 2019 Global Burden of Diseases, Injuries and Risk Factors Study (GBD) identifying it as one of the leading causes of disease worldwide. The post-COVID-19 pandemic era served as a critical inflexion point that dramatically accelerated this burden, exposing and deepening pre-existing vulnerabilities in mental health systems across the globe — the consequences of which continue to reverberate to this day. The widespread enforcement of public health containment measures, including quarantine, isolation, curfews, social distancing, and restrictions on gatherings (4), fundamentally disrupted the social, economic, and academic fabric of daily life.

Remote working, job losses, reduced incomes, and the abrupt shift to virtual learning compounded psychological strain, while fear of infection, bereavement, and prolonged isolation fueled escalating levels of anxiety and depression across populations (5). A landmark cross-sectional population-based online survey conducted across 78 countries at the peak of the pandemic revealed widespread increases in stress and depressive symptoms globally (6), while research in the United States found that the prevalence of depressive symptoms had more than tripled compared to pre-pandemic levels (7). For university students — particularly postgraduate students who were simultaneously managing employment, family responsibilities, and academic demands — the transition to online learning removed critical support structures and intensified an already precarious mental health landscape. Critically, while the acute phase of the pandemic has passed, its psychological legacy persists. Students who experienced heightened distress during this period have carried residual anxiety, diminished motivation, and disrupted coping patterns into the post-pandemic academic environment, contributing to what is now widely recognised as an enduring student mental health crisis that universities across the world, and particularly in low- and middle-income countries, are still struggling to adequately address (8–10).

Stress causes anxiety and depression, the top two contributors of mental disorders globally (8) (9), and while stress can trigger negative emotions and behaviors, different individuals cope differently with some having the ability to effectively cope with adverse emotions and can effectively manage their stress (11). Depending on the coping styles used, an individual can experience favourable or unfavourable consequences on their mental health asserts (12).

Students’ mental well-being is important for good academic performance. Acquiring skills to cope positively with life’s pressures and challenges is a key pillar of mental well-being (13). Understanding coping strategies and their influence on mental well-being will facilitate the provision of tailored mental health services and programmes offered by universities that enhance access to psychosocial support and management. A study conducted at a teaching university hospital in Kenya among medical residents actively managing patients during the COVID-19 pandemic reported the following prevalence of mental disorders among the medical residents: anxiety 51.5%, depression 64.3%, burnout 51.0%, distress 35.4%, and insomnia 40.5% (10).

According to the World Health Organization, stress is a shift that leads to strain on a person’s physical, emotional, or psychological well-being. It is a reaction to any incident or occurrence that requires action, attention, or a response. Stress can manifest in various ways in individuals, for example as concern or worry, jitteriness or an inability to relax, fear, altered sleep patterns, concentration difficulties, and a tendency to use alcohol or drugs that alter one’s mood. When experienced persistently over a long duration, it can cause anxiety, depression, psychosis, and increased use of alcohol and other drugs (14). In another study among 21-40-year-olds who were worried about the impact of the COVID-19 pandemic on the future and the economic challenges posed by the pandemic, increased levels of depression, stress, and anxiety were found among participants who were in the workforce and, as a result, directly affected by potential job furloughs and business closures (15).

Coping refers to the thoughts or behaviours a person uses to mobilise efforts to manage or reduce the stress caused by a situation, challenge, or event. Developing coping skills for stress and life’s challenges is crucial for a person’s well-being, both mental and physical. While there are various approaches people adopt to manage stress, the coping strategies identified are grouped into three as follows: problem-focused, where the individual actively tries to find a solution to the problem encountered; emotion-focused, where one regulates the feelings that arise due to the stressful situation; and problem-avoidant coping, where the person circumvents the problem that is causing stress or is in denial (12) and (16).

In China, a multicenter survey among college students examined how students’ mental health was influenced by their coping style and perceived social support (17). The survey reported poor mental health outcomes among the students who had a passive coping strategy (e.g. smoking and drinking) in comparison to those with active coping strategies. This was more pronounced in developing countries with substantial treatment gaps and inadequate staffing of mental health professionals (18). Postgraduate students face numerous academic and social pressures to be outstanding role models and to succeed in school. However, these pressures are also causing stress and anxiety among university students, and studies confirm that more and more university students are grappling with mental health disorders and engaging in adaptive practices in addition to balancing career expectations, have increased stress and anxiety levels among post-graduate students (8).

In Kenya, for instance, mental health services receive less than 1% of the government’s health budget. The government has not adequately addressed gaps in the provision of services for those with mental illness (10). This situation is further exacerbated by a shortage of mental health workers in health facilities. The availability of mental health services in Kenya is greatly hindered by inadequate resources and a persistent shortage of mental health workers (19). Postgraduate students face numerous academic and social pressures to be outstanding role models and to succeed in school (20–22). However, these pressures are also causing stress and anxiety among university students, and studies confirm that more and more university students are grappling with mental health disorders and engaging in harmful adaptive practices such as substance abuse. Financial challenges and personal and/or family responsibilities, in addition to balancing career expectations, have increased stress and anxiety levels among postgraduate students (8).

The concept of the dual burden is central to understanding the mental health vulnerability of postgraduate students in contexts such as Nairobi. Unlike undergraduate counterparts, postgraduate master’s students in Kenya’s private universities are predominantly working professionals: they hold employment, carry family and parental responsibilities, and self-finance their education, all while meeting the intellectual and performance demands of advanced academic study (20, 23). This simultaneous exposure to occupational stress and academic stress constitutes a dual burden — two intersecting and often compounding sources of psychological demand that together exceed what either domain would impose in isolation. The dual burden concept has been used in global health literature to describe populations exposed to multiple, co-occurring health risks; applied to mental health in higher education, it captures the structural reality that postgraduate students are not simply stressed students but stressed workers, stressed parents, and stressed students simultaneously (23). This compounding exposure elevates their baseline vulnerability to depression, anxiety, and burnout relative to populations carrying only one of these roles (10). Understanding how this dual burden operates and how students cope with it is, therefore, a prerequisite for designing effective institutional responses (24).

Effective coping is essential for responding to various stressors and for improving or maintaining positive mental health (18). However, there has been limited analysis of the association between coping strategies and the mental health outcomes of postgraduate students (20). Evidence from developed countries on coping was more evident during the COVID-19 pandemic, identifying a range of coping strategies (25–27). This study confirms that designing meaningful and effective mental health interventions in universities requires an accurate understanding of how postgraduate students cope with stress and how their strategies affect their mental well-being.

This study confirms that designing meaningful and effective mental health interventions in universities requires an accurate understanding of how postgraduate students cope with stress and how their strategies affect their mental well-being. By focusing on the three main coping strategies and using primary data and descriptive methods in the analysis of the association between coping strategies and mental health outcomes of postgraduate students actively enrolled on Master’s programs. The study sought to answer the following questions:

*RQ_1_* What is the mental health status among postgraduate students?
*RQ_2:_*What are the coping strategies adopted by the postgraduate students?
*RQ_3:_*What is the relationship between the coping strategy adopted and the mental health status of postgraduate students?

## Theoretical and conceptual models

This study is grounded in Lazarus and Folkman’s transactional model of stress and coping, which posits that stress arises from a person’s assessment of a situation or event and that, based on that assessment, coping actions are developed to manage it. Therefore, understanding the intellectual and emotional processes involved in coping may lead to an appreciation of the intricate effects that coping strategies exert on mental well-being (28). The transactional model of stress and coping holds that “psychological stress is a strain between an individual and the conditions they are facing that causes emotional, physical or mental pressure and this condition is judged by the person as surpassing their resources and puts their well-being at risk”. This theory hypothesises that when demands on a person exceed their ability to manage them, stress arises, affecting the person’s mental well-being. Stress can be caused by a multitude of factors arising from one’s obligations. These obligations can be family-related, work-related, financial, health-related, unpleasant childhood experiences, etc., and they can negatively affect the lifestyle the individual was accustomed to (29). This study applied Lazarus and Folkman’s transactional theory of stress and the cognitive appraisal model to examine how postgraduate university students perceive and cope with stress. Using a psychological assessment tool (DASS21) and the Brief COPE inventory, the study assessed relationships between mental well-being and coping strategies among postgraduate students.

**Table 1** presents the operationalisation of the variable indicators that conceptualise the study, guided by Lazarus and Folkman’s Transactional Model of Stress and Coping, which conceptualises coping as a process dependent on individual cognitive appraisal of stressors (28). According to the model, individuals primarily use problem-focused, emotion-oriented, and avoidant coping to manage perceived stress. These coping responses are the primary variables of interest in this study, with a focus on understanding their prevalence and potential implications in the selected population. The model provides a lens for interpreting the influence of different coping strategies on the postgraduate student’s mental health.

**Table 1:** Operationalisation of variables.

Variable	Indicators	Measurement	Data analysis	Supporting literature
Mental health	• Depression • Anxiety • Burnout	Ordinal 5-pt Likert Scale (Quantitative) Qualitative data	Descriptive analysis Correlation analysis Regression analysis	Carver, Scheier, & Weintraub, (1989); Dimitriadou, 2021; Fentahun, et al. (2024).
Problem focused Coping	• Planning • Positive reframing • Active problem solving	Ordinal 5-pt Likert Scale (Quantitative) Qualitative data	Descriptive analysis Correlation analysis Regression analysis	Akim, Miima, and Nthusi (2022); Asnani, et al. (2021)
Emotion oriented Coping	• Involvement in religious activities • Venting • Humor	Ordinal 5-pt Likert Scale (Quantitative) Qualitative data	Descriptive analysis Correlation analysis Regression analysis	Akim, Miima, and Nthusi, (2022); Frei, Sazhin, Fick, and Yap (2021)
Avoidant Coping	• Self-blame • Disengagement • Denial	Ordinal 5-pt Likert Scale (Quantitative) Qualitative data	Descriptive analysis Correlation analysis Regression analysis	Akim, Miima, and Nthusi, (2022); Bartone and Homish (2020)

**Table 2:** Respondent profile by master’s programme — proportionate stratified sample.

Masters programme	Population (N)	Sample (n)	% of total
Master’s in Business Administration	199	79	32.2%
Master of Management in Agribusiness	42	17	6.9%
Master’s in Healthcare Management	83	33	13.5%
Master of Science in Development Finance	122	49	20.0%
Master’s in Public Policy and Management	54	22	9.0%
Master of Commerce	112	45	18.4%
Total	612	245	100.0%

## Methods

### Study design

A descriptive quantitative cross-sectional survey design was adopted, given the various categories of the programs undertaken by the study participants. It also helped with establishing relationships and nature of variables are contextualised and described (30), and data were collected and analysed (31, 32). The various postgraduate programs provided key sections for the study design. Additionally, descriptive designs help describe the relationships between the dependent and independent variables. In this study, quantitative data were collected and analysed to describe the phenomena and establish any correlations. Using a descriptive design, the researcher sought to examine postgraduate students’ mental well-being, the coping strategies they adopted, and the associations between these strategies and students’ mental well-being.

### Population and sampling

The target population was postgraduate students at Strathmore University who were actively enrolled in an academic program and are working at the same time. This study adopted a random stratified sampling design, in which all master’s students who are working concurrently while studying. Within each stratum, participants were randomly selected, reducing sampling bias and enhancing the study’s overall validity. Using records obtained from the school administration, the study included 612 postgraduate master’s students across 9 master’s programs at Strathmore University, with these programs serving as the strata for the sample frame in a proportionate manner. formed the strata or subgroups from which the random sample was drawn, and the sample size of 242 students was included in this study. The sample size for the study is determined using the Yamane formula.

### Data collection instruments and procedure

In this study, a questionnaire was used to collect data and was sent electronically to the study population. The questionnaire collected demographic information on students’ personal, work, and academic details, as well as data from the Depression, Anxiety, and Stress Scale (DASS-2) and the Coping Orientation to Problems Experienced (COPE) inventory. These two questionnaires measured postgraduate students’ mental health status and the coping strategies they employed during stress, respectively. The DASS-2 is a self-reported questionnaire designed to assess the levels of depression, anxiety, and stress experienced by an individual. It consists of 42 items across three 14-item subscales and was developed by (33). The COPE inventory is a self-reported questionnaire that assessed the ways in which an individual COPE with stress and challenging events. It contains items that assess different coping strategies. It was developed by (34). The Coping Orientation to Problem Experienced was conceptualised using a problem-focused approach, emotion-focused and avoidant coping strategies. The 28 Likert statements were interpreted using the following key: 4.20 and above fully agree, 3.50- 4.19 = agree, 2.50 -3.49 = neither agree nor disagree, 1.50 – 2.49 = disagree, less than 1.49 = fully disagree. The questionnaires were explained to the students with an option of either physical or online administration. The questionnaires were explained to the students, with an option of either physical or online administration.

### Data analysis and presentation

Data were once collected, cleaned, coded, and entered SPSS (version 23) for analysis. The background section focused on the profile of the respondents’ demographic profiles such as age, gender, type of master’s program, year of study, family background as well as their characteristics of mental health service utilization among students which were analyzed using descriptive statistics. Descriptive analysis was used to characterise the study population. Inferential statistics (multiple regression and correlation analysis) were used to assess associations among the study variables. The study collected research data using the Depression, Anxiety and Stress Scale (DASS2) and COPE (Coping Orientation to Problem Experienced) inventory. The various responses obtained were analyzed using descriptive measures such as means and standard deviation.

### Ethical consideration

This study was conducted in conformity with the ethical standards and procedures. Ethical approval was sought from the Strathmore University Ethics and Research Committee (SU-ISERC1992/24) and the National Commission for Science, Technology and Innovation (NACOSTI 383882). The respondent’s information sheet for consent was in English in line with the language used for data collection. All data collected were utilised in this study’s analysis, with confidentiality maintained by using codes rather than participants’ names as identifiers.

## Findings

### Background information

This section of the findings focused on the respondents’ profiles as background information.

The results demonstrated that 27% of respondents were in Year 1 and 39% in Year 2 of their studies. This revealed that participants included in the survey have varying periods of study, thus have been exposed to the master’s programme differently, which can help in identifying how they have been coping with any mental health issues during this period. **Table 3** presents a consolidated background profile of the 245 respondents across three dimensions: year of study, age bracket, and master’s programme, disaggregated by gender.

**Table 3:** Background profile of respondents by gender — year of study, age bracket, and master’s programme.

Category	Male (n)	Male (%)	Female (n)	Female (%)	Row total (n)
A: Year of Study					
Year 1	28	42.4%	38	57.6%	66 (27%)
Year 2	36	37.5%	60	62.5%	96 (39%)
Year 3	28	42.4%	38	57.6%	66 (27%)
Year 4	8	47.1%	9	52.9%	17 (7%)
Year Total	100	40.8%	145	59.2%	245 (100%)
B: Age Bracket					
23–27 years	4	10.8%	33	89.2%	37 (15.1%)
28–32 years	28	40.6%	41	59.4%	69 (28.2%)
33–37 years	26	42.6%	35	57.4%	61 (24.9%)
38–42 years	22	50.0%	22	50.0%	44 (18.0%)
43–49 years	14	58.3%	10	41.7%	24 (9.8%)
50 years and above	6	60.0%	4	40.0%	10 (4.1%)
Age Total	100	40.8%	145	59.2%	245 (100%)
C: Master’s Programme					
Master’s in Business Administration	38	48.1%	41	51.9%	79 (32.2%)
Master of Management in Agribusiness	8	47.1%	9	52.9%	17 (6.9%)
Master’s in Healthcare Management	15	30.6%	34	69.4%	49 (20.0%)
Master of Science in Development Finance	14	42.4%	19	57.6%	33 (13.5%)
Master’s in Public Policy and Management	16	35.6%	29	64.4%	45 (18.4%)
Master of Commerce	9	40.9%	13	59.1%	22 (9.0%)
Programme Total	100	40.8%	145	59.2%	245 (100%)

Across all three sections, female students consistently outnumber male students, with an overall gender split of 59.2% female (n=145) and 40.8% male (n=100), reflecting the broader trend of rising female enrolment in postgraduate education at Kenyan private universities. With respect to year of study, the majority of respondents were in Year 2 (39%, n=96), representing students within the standard programme schedule, while Years 3 and 4 combined account for 34% of the cohort — students who had exceeded the standard two-year duration, either with outstanding coursework or ongoing dissertations. The age distribution reveals a predominantly mid-career cohort, with over two-thirds of respondents (68.2%) aged 23 to 37 years. The 23–27 bracket is overwhelmingly female (89.2%), consistent with younger students enrolling directly or shortly after undergraduate study, while the gender balance progressively shifts with age — the 43–49 and 50+ brackets show a slight male majority, reflecting senior male professionals pursuing postgraduate credentials later in their careers. By programme, the master’s in business administration accounts for the largest share of respondents (32.2%, n=79), followed by Healthcare Management (20.0%) and Public Policy and Management (18.4%). Healthcare Management records the highest female concentration (69.4%), while Agribusiness and MBA show the most balanced gender distributions. Taken together, the profile confirms a diverse, working-age, predominantly female postgraduate cohort navigating study across different stages of their academic and professional lives (**Table 2**).

The findings further focused on the respondents’ background, particularly their family status, living status, employment status, and payment of their programme fees. The summary of the results is shown in **Table 4**.

**Table 4:** Family background of respondent.

Demographics information		Frequency	Percent
Family Status	Unmarried	50	20.4
	Married	42	17.1
	Single parent/separated	118	48.2
	Widowed	34	13.9
	Total	244	99.6
Living status	Alone	66	26.9
	Family	158	64.5
	Friends	20	8.2
	Total	244	99.6
Employment status	Self-employed	45	18.4
	Employed	165	67.3
	Unemployed	32	13.1
	Total	242	98.8
Fee Payment	Self-sponsored	169	69.0
	Scholarship	55	22.4
	Total	245	100.0

**Table 4** presents the family background characteristics of the 245 respondents. With regard to family status, the majority of the students were single parents (48.2%, n=118), followed by unmarried students (20.4%, n=50), married students (17.1%, n=42), and widowed students (13.9%, n=34). This suggests that a significant proportion of Masters students at Strathmore University carry parental responsibilities alongside their studies, which may have implications for their mental health and academic progression. In terms of living arrangements, most respondents lived with family (64.5%, n=158), while 26.9% (n=66) lived alone and 8.2% (n=20) lived with friends. Regarding employment status, the majority were employed (67.3%, n=165), with 18.4% (n=45) self-employed and 13.1% (n=32) unemployed, indicating that most students were balancing full-time or part-time work commitments alongside their studies. Finally, with respect to fee payment, 69.0% (n=169) were self-sponsored and 22.4% (n=55) were on scholarships, reflecting a high degree of financial self-reliance among the student population. Taken together, these findings highlight the complex socio-economic circumstances of Master’s students at Strathmore University — many of whom are single parents, employed, and self-financing their education — factors that collectively contribute to the unique mental health pressures experienced within this postgraduate cohort.

The study further, reviewed the status of usage of mental health support services, and a summary of the responses obtained is shown in **Table 3**. Analysis showed that the majority of respondents, 62% (n = 153) of the students, were not utilising the university’s mental health support services, with only 37% using them. This indicated a very low uptake of the services among the master’s students. The research showed that 58% (n =141) of the students felt the mental health services were not a good fit for them, while 18% indicated they can be excellent. This revealed diverse opinions among the students on the utilization of mental services. Results indicated that 4% of respondents accessed mental health services through colleague referrals (**Table 5**).

**Table 5:** Utilisation of mental health services.

Statement	Statement on mental service access mode	Frequency	Percent
Utilization of mental health support services offered by the university	Yes	91	37.1
	No	153	62.4
	Total	244	99.6
Description of mental health services	No response	12	4.9
	Excellent	44	18.0
	Good	37	15.1
	Not a good fit for me	141	57.6
	Total	234	95.5
Access to mental health services	By one-self	3	1.2
	Referral	9	3.7
	Total	12	4.9
Importance of Mental Health Support services	Yes	233	95.1
	No	12	4.9
	Total	245	100.0

## Results on depression, anxiety and stress scale

**Table 6a** presents the results of the Depression, Anxiety and Stress Scale (DASS-2) across 245 Masters students at Strathmore University. The mean scores for all items ranged between 2.23 and 2.73, falling within the 2.5–3.49 range (applicable to a considerable degree) or just below it (applicable to some degree), indicating moderate but consistent psychological distress across the cohort.

**Table 6A:** Results on depression, anxiety and stress scale.

Rank	DASS-2 item	N	Mean	Std. deviation
1	I was worried about a situation in which I might panic and make a fool of myself	244	2.73	0.99315
2	I found myself getting agitated	244	2.68	1.00349
3	I found it difficult to relax	243	2.65	0.96481
4	I found it difficult to work up the initiative to do things	243	2.64	0.95807
4	I tended to over-react to situations	244	2.64	0.94833
6	I found it hard to wind down	244	2.62	0.85902
7	I was intolerant of anything that kept me from getting on with what I was doing	242	2.60	0.93817
8	I felt I was using a lot of nervous energy	244	2.54	0.99088
8	I felt I was close to panic	245	2.54	0.97695
8	I was unable to become enthusiastic about anything	241	2.54	1.04458
11	I felt scared without any good reason	244	2.52	1.04005
12	I felt I had nothing to look forward to	244	2.50	1.01226
12	I felt rather touchy	243	2.50	0.96368
14	I couldn’t seem to experience any positive feelings at all	245	2.45	0.99735
14	I was aware of the action of my heart in the absence of physical exertion (increased heart rate, heartbeat missing a beat)	244	2.45	1.06285
16	I felt down-hearted and blue	244	2.44	0.97337
16	I felt I wasn’t worth as a person	244	2.44	1.05033
18	I experienced breathing difficulty (rapid breathing, breathlessness) in the absence of any physical activity or exertion	244	2.38	1.00524
18	I experienced trembling (e.g. in the hands)	242	2.38	1.07600
20	I was aware of dryness of my mouth	243	2.29	1.05203
21	I felt that life was meaningless	245	2.23	1.01277

**Table 6B:** DASS-21 subscale mean scores by gender and age bracket (n=245).

Group	Stress mean	Anxiety mean	Depression mean	Overall interpretation
A: DASS-21 Subscale Scores by Gender				
Male (n=100)	2.65	2.44	2.31	Some degree
Female (n=145)	2.57	2.54	2.43	Considerable
Overall (n=245)	2.60	2.50	2.38	Considerable
B: DASS-21 Subscale Scores by Age Bracket				
23–27 years (n=37)	2.52	2.71	2.35	Considerable
28–32 years (n=69)	2.68	2.55	2.38	Considerable
33–37 years (n=61)	2.72	2.50	2.40	Considerable
38–42 years (n=44)	2.61	2.46	2.42	Some degree
43–49 years (n=24)	2.48	2.38	2.44	Some degree
50 years and above (n=10)	2.41	2.28	2.48	Some degree
Overall (n=245)	2.60	2.50	2.38	Considerable

The highest mean score was recorded for “I was worried about a situation in which I might panic and make a fool of myself” (M = 2.73, SD = 0.99), suggesting that social anxiety and fear of public embarrassment were the most prominently experienced symptoms. This was closely followed by “I found myself getting agitated” (M = 2.68, SD = 1.00) and “I found it difficult to relax” (M = 2.65, SD = 0.96), both indicative of elevated stress responses.

Items related to depression were also notable. “I felt I had nothing to look forward to” (M = 2.50), “I was unable to become enthusiastic about anything” (M = 2.54), and “I felt I wasn’t worth as a person” (M = 2.44) reflect a discernible degree of low mood, loss of motivation, and diminished self-worth among respondents. The lowest mean was recorded for “I felt that life was meaningless” (M = 2.23), though its presence above the lower threshold still warrants attention.

Standard deviations across items were generally moderate (ranging from 0.86 to 1.06), suggesting a reasonably consistent experience of these symptoms across the student population rather than isolated cases. Overall, the findings indicate that Masters students at Strathmore University experience measurable levels of depression, anxiety, and stress, with anxiety-related symptoms — particularly panic anticipation, agitation, and difficulty relaxing — being the most prominent.

Section A reveals notable gender differences across all three DASS-21 subscales. Male respondents recorded a higher stress mean (M = 2.65) compared to females (M = 2.57), consistent with evidence that men in professional settings internalise occupational and academic pressure as stress rather than anxiety. Conversely, female respondents reported higher anxiety (M = 2.54 vs M = 2.44) and depression (M = 2.43 vs M = 2.31) means, a pattern well-documented in the mental health literature where women are more likely to experience and report affective and anxiety-related symptoms. Despite these differences, both groups fall predominantly within the considerable degree range, confirming that psychological distress is pervasive across genders.

Section B reveals a clear age-related gradient in the pattern and intensity of distress. The youngest bracket (23–27 years) records the highest anxiety mean (M = 2.71), reflecting the performance pressure and identity uncertainty characteristic of early postgraduate experience. Stress scores peak in the 33–37 bracket (M = 2.72), corresponding to the period of greatest dual burden — when professional responsibilities, family obligations, and academic demands are simultaneously at their most intense.

### Problem-focused coping strategy

The first indicator in the COPE inventory reviewed the problem-focused coping strategy and the analysis of the various items is shown in **Table 7**.

**Table 7:** Results on problem-focused coping strategy (ranked by mean score, highest to lowest).

Rank	Problem-focused coping item	N	Mean	Std. deviation
1	I’ve been looking for something good in what is happening	244	3.59	1.10929
2	I’ve been concentrating my efforts on doing something about the situation I’m in	245	3.52	1.15433
3	I’ve been thinking hard about what steps to take	244	3.51	1.13481
4	I’ve been trying to see it in a different light, to make it seem more positive	244	3.50	1.13492
5	I’ve been trying to come up with a strategy about what to do	245	3.48	1.21319
6	I’ve been taking action to try to make the situation better	244	3.46	1.15931
7	I’ve been getting help and advice from other people	243	3.41	1.25419
8	I’ve been trying to get advice or help from other people about what to do	243	3.37	1.23427

**Table 7** presents the results for the problem-focused coping strategy items from the COPE inventory, ranked from highest to lowest mean score. The highest-rated item was “I’ve been looking for something good in what is happening” (M = 3.59, SD = 1.11), indicating that positive reframing was the most commonly adopted problem-focused strategy among respondents, which falls within the agreed range. This was closely followed by “I’ve been concentrating my efforts on doing something about the situation I’m in” (M = 3.52, SD = 1.15) and “I’ve been thinking hard about what steps to take” (M = 3.51, SD = 1.13), both of which reflect active cognitive engagement with the stressor. The remaining items — including trying to see the situation more positively (M = 3.50), coming up with a strategy (M = 3.48), and taking direct action (M = 3.46) — also fell within or at the boundary of agreement, suggesting that students generally engaged with problem-focused coping at a moderate to agreeable level.

### Emotion-focused coping strategy

**Table 8** presents the results for the emotion-focused coping strategy items from the COPE inventory, ranked from highest to lowest mean score.

**Table 8:** Results on emotion-focused coping strategy (ranked by mean score, highest to lowest).

Rank	Emotion-focused coping item	N	Mean	Std. deviation
1	I’ve been learning to live with it	243	3.58	1.14816
2	I’ve been accepting the reality of the fact that it has happened	243	3.52	1.20369
3	I’ve been praying or meditating	243	3.51	1.18304
4	I’ve been getting comfort and understanding from someone	243	3.47	1.21386
5	I’ve been trying to find comfort in my religion or spiritual beliefs	244	3.39	1.21755
6	I’ve been expressing my negative feelings	243	3.36	1.23672
7	I’ve been blaming myself for things that happened	245	3.35	1.25092
8	I’ve been criticising myself	244	3.34	1.22519
8	I’ve been getting emotional support from others	243	3.34	1.26021
10	I’ve been making jokes about it	245	3.25	1.24517
11	I’ve been saying things to let my unpleasant feelings escape	244	3.18	1.24071
12	I’ve been making fun of the situation	245	3.08	1.31256

The highest-rated strategy was “I’ve been learning to live with it” (M = 3.58, SD = 1.15), indicating that acceptance-based coping was the most predominant emotion-focused strategy among respondents, falling within the agreed range. This was closely followed by “I’ve been accepting the reality of the fact that it has happened” (M = 3.52, SD = 1.20) and “I’ve been praying or meditating” (M = 3.51, SD = 1.18), both of which also fell within the agreed range and suggest that spiritual and acceptance-oriented coping mechanisms play a significant role in how Masters students at Strathmore University manage emotional distress.

Strategies related to seeking comfort and understanding from others (M = 3.47) and finding solace in religion or spiritual beliefs (M = 3.39) were also prominent. In contrast, strategies involving self-criticism, such as “I’ve been criticising myself” (M = 3.34) and “I’ve been blaming myself for things that happened” (M = 3.35), fell within the neither agreed nor disagreed range, yet their relatively elevated scores indicate that self-blame remains a notable, if moderate, coping response. The lowest-ranked strategies were “I’ve been making fun of the situation” (M = 3.08) and “I’ve been saying things to let my unpleasant feelings escape” (M = 3.18), suggesting that humour and emotional venting were the least commonly employed emotion-focused coping mechanisms within this cohort. Overall, the findings highlight a tendency towards internalised and spiritually oriented coping strategies over expressive or avoidant approaches.

### Avoidant-focused coping strategy

The third item assessed in the COPE inventory was the various avoidant-focused strategies, and the findings are shown in **Table 9**.

**Table 9:** Results on avoidant-focused coping strategy (ranked by mean score, highest to lowest).

Rank	Avoidant-focused coping item	N	Mean	Std. deviation
1	I’ve been doing something to think about it less, such as going to movies, watching TV, reading, daydreaming, sleeping, or shopping	242	3.46	1.27238
2	I’ve been turning to work or other activities to take my mind off things	244	3.41	1.09023
3	I’ve been giving up the attempt to cope	243	3.11	1.34840
4	I’ve been refusing to believe that it has happened	243	3.06	1.23328
4	I’ve been using alcohol or other drugs to help me get through it	244	3.06	1.37765
6	I’ve been saying to myself “this isn’t real”	244	3.00	1.34714
7	I’ve been giving up trying to deal with it	244	2.98	1.30187
8	I’ve been using alcohol or other drugs to make myself feel better	245	2.94	1.39848

The highest-rated avoidant strategy was “I’ve been doing something to think about it less, such as going to movies, watching TV, reading, daydreaming, sleeping, or shopping” (M = 3.46, SD = 1.27), suggesting that distraction through leisure activities was the most commonly employed avoidant coping mechanism. This was closely followed by “I’ve been turning to work or other activities to take my mind off things” (M = 3.41, SD = 1.09), indicating that redirecting attention to productive or recreational tasks was also a prominent avoidant response among the students.

In contrast, substance-related avoidance strategies ranked the lowest. “I’ve been using alcohol or other drugs to make myself feel better” recorded the lowest mean (M = 2.94, SD = 1.40), followed by “I’ve been giving up trying to deal with it” (M = 2.98, SD = 1.30). These findings suggest that while students moderately resort to behavioural distraction as an avoidant coping mechanism, they are less inclined to resort to substance use or complete disengagement. Nonetheless, the presence of these responses — even at moderate levels — warrants attention from a mental health and student welfare perspective.

**Table 10a** summarises the mean scores across the three COPE subscales, and **Table 10b** presents the indicative subgroup differences in dominant coping strategy by sex, age bracket, and programme stream.

**Table 10A:** COPE subscale averages — overall summary (n=245).

COPE subscale	Item range (M)	Subscale mean	SD (approx.)	Likert interpretation
Problem-Focused Coping	3.37 – 3.59	3.49	1.16	Agree (boundary)
Emotion-Focused Coping	3.08 – 3.58	3.37	1.22	Neither agree/disagree to Agree
Avoidant Coping	2.94 – 3.46	3.13	1.31	Neither agree nor disagree

**Table 10B:** Indicative COPE subgroup differences by sex, age, and programme stream.

Dimension	Subgroup	Dominant coping strategy	Alignment with DASS-21 profile
Sex	Female (n=145)	Emotion-focused (acceptance, prayer, social support)	Higher anxiety (M = 2.54) and depression (M = 2.43)
	Male (n=100)	Problem-focused (planning, active coping)	Higher stress (M = 2.65); internalised occupational pressure
Age	23–27 years (n=37)	Emotion-focused (social support, acceptance)	Highest anxiety (M = 2.71); identity uncertainty
	33–37 years (n=61)	Problem-focused (planning, active coping)	Peak stress (M = 2.72); dual burden at its most intense
	43+ years (n=34)	Avoidant + acceptance-based emotion-focused	Lower overall distress; experienced coping repertoire
Programme Stream	Healthcare Management (n=49)	Emotion-focused (highest alignment)	Predominantly female (69.4%); empathetic regulatory norms
	MBA (n=79)	Broad distribution across all three types	Most gender-balanced and occupationally diverse cohort
	Development Finance (n=33)	Problem-focused (planning, instrumental support)	Technically demanding programme; analytical orientation

**Table 10C:** Spearman’s rank correlation matrix — coping strategies and mental health.

Variable	Statistic	Mental health	Problem-focused coping	Emotion-focused coping	Avoidant coping
Mental Health	Correlation (r)	1.000			
	Sig. (2-tailed)	—			
	N	245			
Problem-Focused Coping	Correlation (r)	.302**	1.000		
	Sig. (2-tailed)	.000	—		
	N	245	245		
Emotion-Focused Coping	Correlation (r)	.494**	.726**	1.000	
	Sig. (2-tailed)	.000	.000	—	
	N	245	245	245	
Avoidant Coping	Correlation (r)	.559**	.509**	.664**	1.000
	Sig. (2-tailed)	.000	.000	.000	—
	N	245	245	245	245

** Correlation is significant at the 0.01 level (2-tailed). N = 245 for all variables.

Subgroup coping patterns are indicative and based on item-level mean distributions; formal inferential testing of differences in coping strategies by subgroup was not conducted. The rank order of subscale averages — problem-focused highest (M = 3.49), emotion-focused intermediate (M = 3.37), avoidant lowest (M = 3.13) — indicates that students nominally favour active engagement with stressors, yet it is emotion-focused and avoidant.

### Correlation analysis

Spearman’s rank correlation analysis was conducted to assess the bivariate relationships between the three coping strategy dimensions (problem-focused, emotion-focused, and avoidant) and the mental health of postgraduate students (n=245). The Spearman’s rho (r) coefficient was used, given the ordinal nature of the Likert-scale data. Correlation coefficients range from -1.0 to +1.0, with values closer to +1 or -1 indicating stronger relationships; p <.05 denotes statistical significance.

The results of the correlation analysis are presented in **Table 10c**. Problem-focused coping demonstrated a weak but statistically significant positive relationship with mental health (r = .302**, p = .000 <.05), suggesting that higher levels of active, solution-oriented coping are modestly associated with improved mental health outcomes. Emotion-focused coping showed a moderate positive and significant correlation with mental health (r = .494**, p = .000 <.05), indicating that strategies such as acceptance, prayer, meditation, and emotional expression are more strongly associated with students’ psychological well-being. Avoidant coping recorded the strongest correlation with mental health among the three predictors (r = .559**, p = .000 <.05), reflecting a moderate-to-strong positive relationship and signalling that distraction-based and disengagement strategies are most closely aligned with mental health outcomes in this population.

The inter-predictor correlations also reveal noteworthy relationships. Emotion-focused and avoidant coping share a strong positive correlation (r = .664**, p = .000), while problem-focused and avoidant coping are moderately correlated (r = .509**, p = .000), and problem-focused and emotion-focused coping are also significantly related (r = .726**, p = .000). These inter-correlations indicate that students who employ one category of coping are also likely to use others, consistent with the multi-dimensional nature of stress management. While these intercorrelations are moderate to strong, multicollinearity was assessed in the subsequent regression analysis.

### Regression analysis

Multiple linear regression analysis was conducted to examine the joint predictive effect of the three coping strategy dimensions on the mental health of postgraduate students at Strathmore University. The coping strategies (problem-focused, emotion-focused, and avoidant) served as the independent variables, and mental health as measured by the DASS-2 served as the dependent variable. The analysis was presented across three components: the model summary (**Table 11**), the ANOVA (**Table 12**), and the regression coefficients (**Table 13**).

**Table 11:** Model summary — regression of coping strategies on mental health.

Model	R	R²	Adjusted R²	Std. error of the estimate
1	.634^a^	.402	.395	.48755

^a^Predictors: (Constant), Avoidant Coping, Problem-Focused Coping, Emotion-Focused Coping. Dependent Variable: Mental Health.

**Table 12:** ANOVA — test of overall regression model significance.

Model	Source of variation	Sum of squares (SS)	Df	Mean square (MS)	F	Sig.
1	Regression	38.502	3	12.834	53.991	.000^b^
	Residual (Error)	57.288	241	.238		
	Total	95.790	244			

^a^ Dependent Variable: Mental Health. ^b^ Predictors: (Constant), Avoidant Coping, Problem-Focused Coping, Emotion-Focused Coping.

**Table 13:** Regression coefficients — individual effects of coping strategies on mental health.

Model	Predictor variable	Unstandardized coefficients		Standardized coefficients (β)	T	Sig.
		B	Std. error	Beta (β)	T-value	P-value
1	(Constant)	1.068	.156	—	6.857	.000
	Problem-Focused Coping	-.189	.062	-.235	-3.062	.002
	Emotion-Focused Coping	.307	.076	.356	4.071	.000
	Avoidant Coping	.344	.047	.486	7.264	.000

Dependent Variable: Mental Health. B, unstandardized coefficient; SE, standard error; Beta (β), standardized coefficient. *p <.05, **p <.01, ***p <.001.

### Model summary

**Table 11** presents the overall fit of the regression model. The multiple correlation coefficient R = .634 indicates a strong positive association between the three coping strategy predictors combined and mental health outcomes. The coefficient of determination, R² = .402, indicates that coping strategies collectively explain 40.2% of the variance in postgraduate students’ mental health, with the adjusted R² = .395 confirming this result after accounting for model complexity and sample size. The remaining 59.8% of variance is attributable to factors not captured in the model, such as socioeconomic conditions, individual resilience, or external support systems. The standard error of the estimate (.488) indicates the average margin by which the model’s predictions deviate from observed mental health scores, reflecting acceptable model precision.

### ANOVA — model significance

The ANOVA in **Table 12** was used to test the overall statistical significance of the regression model. The regression sum of squares (SS = 38.502, df = 3, MS = 12.834) represents the portion of total variance in mental health that is explained by the three coping predictors. The residual sum of squares (SS = 57.288, df = 241, MS = .238) captures the unexplained variance. The total sum of squares is 95.790 (df = 244). Dividing the regression mean square by the residual mean square yields an F-value of 53.991, which substantially exceeds the F-critical value of 3.902 at df (3, 241) and the.05 significance level. The associated significance value of p = .000 <.05 leads to rejection of the null hypothesis that none of the coping strategies predict mental health. The model is therefore statistically significant and well-fitted for further coefficient-level interpretation.

### Regression coefficients

**Table 13** presents the individual contribution of each coping strategy predictor to mental health outcomes. The constant (B = 1.068, SE = .156, t = 6.857, p = .000) represents the estimated baseline mental health score when all predictor values are held at zero. Each coefficient is interpreted below.

Problem-Focused Coping (B = -.189, SE = .062, Beta = -.235, t = -3.062, p = .002): Problem-focused coping exerted a negative and statistically significant effect on mental health. For each 1-unit increase in problem-focused coping, mental health scores decreased by 0.189 units, holding all other variables constant. The standardised beta of -.235 indicates a small-to-moderate negative effect. This finding is counterintuitive relative to established coping theory but reflects the contextual reality that many stressors faced by Strathmore’s postgraduate students — financial pressure, parental responsibilities, and work demands — are structural in nature and resistant to direct problem-solving, making active confrontation more distressing rather than relieving.

Emotion-Focused Coping (B = .307, SE = .076, Beta = .356, t = 4.071, p = .000): Emotion-focused coping demonstrated a positive and statistically significant effect on mental health. Each unit increase in emotion-focused coping was associated with a.307 unit improvement in mental health, with a standardised beta of.356 indicating a moderate positive effect. Strategies such as acceptance, prayer, meditation, and seeking emotional understanding appear particularly beneficial for this cohort, aligning with the study’s theoretical framework, which recognises emotion-focused coping as adaptive when stressors are appraised as uncontrollable.

Avoidant Coping (B = .344, SE = .047, Beta = .486, t = 7.264, p = .000): Avoidant coping emerged as the strongest and most statistically significant predictor of mental health in the model, with the highest standardised beta coefficient (Beta = .486). Each unit increase in avoidant coping was associated with a.344 unit improvement in mental health. This positive effect reflects the beneficial role of behavioural disengagement strategies — such as leisure activities, self-distraction, and temporary withdrawal from stressors — in providing psychological relief. The overall regression equation derived from these findings can be expressed as:


$$Mental\;Health\;=\;1.068\;+\;\left(-.189\;x\;Problem-Focused\;Coping\right)\;+\;\left(.307\;x\;Emotion-Focused\;Coping\right)\;+\;\left(.344\;x\;Avoidant\;Coping\right)$$

In summary, the regression analysis confirms that coping strategies are significant predictors of postgraduate student mental health, collectively accounting for 40.2% of its variance (R² = .402, F = 53.991, p = .000). Among the three predictors, avoidant coping exerts the greatest influence, followed by emotion-focused coping, while problem-focused coping has a small but significant negative effect. These findings have direct implications for how mental health interventions should be designed and targeted within the postgraduate university context.

## Discussion

The findings of this study are best understood through the lens of the dual burden that characterises postgraduate study at a private Kenyan university. Unlike traditional student populations whose stress exposure is largely bounded by academic demands, the cohort studied here simultaneously carries the weight of professional employment (67.3% employed or self-employed), parental and family responsibility (48.2% single parents, 64.5% living with family), and financial self-reliance (69.0% self-sponsored). These are not peripheral stressors sitting alongside academic life — they are structural features of the students’ existence that render academic demands one layer of pressure within a multi-layered burden. The dual burden framework, as applied here, helps explain why mental health distress in this cohort is broadly distributed, why conventional coping prescriptions (particularly problem-focused strategies) may paradoxically exacerbate distress, and why emotion-focused and avoidant strategies prove protective: students facing stressors that are structurally determined and largely uncontrollable are better served by strategies that regulate emotional response than by strategies that presuppose the stressor can be solved (35). The discussion below interprets the mental health and coping findings in light of this dual burden context.

### Mental health status of postgraduate master’s students

The first research question sought to establish the mental health status of postgraduate students at Strathmore University as measured by the Depression, Anxiety and Stress Scale (DASS-2). The findings revealed moderate but consistent psychological distress across the cohort, with all 21 items recording mean scores between 2.23 and 2.73, spanning the applicable to some degree (1.50-2.49) and applicable to a considerable degree (2.50-3.49) bands of the scale. Standard deviations ranged narrowly from 0.86 to 1.07, indicating that this distress was not limited to isolated individuals but was broadly shared across the student population. These findings are consistent with (23), who documented that graduate students report anxiety and depression at rates significantly exceeding the general population, and which had been confirmed high co-occurrence of depression, anxiety and stress among university students (8).

Anxiety and stress symptoms were the most prominent features of the mental health profile. The highest-ranked item, I was worried about a situation in which I might panic and make a fool of myself (M = 2.73, SD = 0.99), reflects elevated social evaluation anxiety consistent with the performance pressures of postgraduate study. This was closely followed by indicators of sustained stress arousal: getting agitated (M = 2.68), difficulty relaxing (M = 2.65), over-reacting to situations (M = 2.64), and inability to wind down (M = 2.62). Chronic academic stress and the inability to disengage from academic demands are central impediments to student well-being, a pattern clearly mirrored here (36, 37). Somatic anxiety symptoms were also evident, including feeling close to panic (M = 2.54), unexplained fear (M = 2.52), heart palpitations (M = 2.45), and breathing difficulties (M = 2.38), which extend the distress profile beyond cognitive symptoms into physiological manifestations, as similarly documented (9, 38).

Depression-related symptoms, while scoring below the considerable degree threshold, were nonetheless present across the cohort. Items reflecting loss of enthusiasm (M = 2.54), low self-worth (M = 2.44), low mood (M = 2.44), and a sense of meaninglessness in life (M = 2.23) point to subclinical depressive features warranting institutional attention. These patterns are consistent with findings from the African higher education context, where Fentahun et al. (2024) (39) reported a pooled depression prevalence of 33.8% among Ethiopian university students, and Ndegwa et al. (2020) (40) documented significant depression rates among university students in Nairobi. The presence of these depressive indicators alongside anxiety and stress symptoms confirms a multi-dimensional distress profile that aligns with the dual-burden framing of the study (41).

The socio-demographic context of the respondents provides important explanatory depth. The majority were single parents (48.2%), employed (67.3%), and self-sponsored (69.0%), a configuration that substantially amplifies baseline stress vulnerability. As Lazarus and Folkman (28) posit, stress arises when demands are perceived to exceed available resources, and for students simultaneously managing parenthood, employment, financial responsibility, and academic rigour, this threshold is readily crossed. Similarly, other studies (24) noted that role conflict and perceived burden are key mediators of mental health deterioration among students. Furthermore, similar documented high rates of anxiety (51.5%), depression (64.3%), and burnout (51.0%) among postgraduate residents in Kenya, providing a proximal benchmark that reinforces the severity of distress within this population (10).

At the institutional level, 57.6% of students who had accessed mental health services described them as not a good fit, despite 95.1% affirming their importance. This gap between demand and perceived relevance signals a fundamental mismatch between available support and student needs, echoing findings from (18, 42), who identified structural and contextual inadequacies in university mental health provision across developing-country settings. Kenya’s allocation of less than 1% of its national health budget to mental health services (10, 19) further constrains the capacity for institutional response. These findings collectively call for the development of targeted, contextually grounded, and structurally integrated mental health interventions that reflect the distinctive socioeconomic and academic realities of Kenyan postgraduate students (43).

### Problem-focused coping strategy

The first objective of the study was to examine the relationship between the problem-focused coping strategy and the mental health of postgraduate students at Strathmore University. Based on the correlation analysis, the relationship was significant but negative. These findings imply that problem-focused coping strategies, which are mostly action-oriented, exacerbate mental health illnesses among postgraduate students (44). These findings are consistent with (45), which found that, in some instances, direct coping strategies offer only short-term benefits and cause significant long-term harm. This is particularly pertinent in the present post-COVID-19 study context: while COVID-19 is no longer an active stressor for students, its residual effects on academic systems, financial stability, and mental health infrastructure continue to shape the conditions under which students cope (46). Unlike pandemic-specific findings, the constraints faced by Strathmore’s postgraduate students — financial pressures, role overload, and structural barriers to change — are enduring features of their lives that problem-focused strategies cannot easily resolve, making sustained effort toward uncontrollable stressors psychologically costly rather than relieving (47).

These findings that problem-focused coping has negative impacts on students’ mental health go against the expectations of the stress and coping theory, which stresses that dealing directly with a stressor is the most effective way to address it. The study was, however, unique in that it sought to identify coping strategies used by poor students. The finding that problem-focused coping strategies have negative effects on an individual’s mental health also contradicts expectations made by researchers (13, 27, 48–50) who all revealed a positive effect of problem-focused coping on an individual’s mental health.

In a study among Spanish undergraduate students who used problem-solving practices, including cognitive restructuring, activity pacing, and visualisation, all reported improved health-related quality of life (51, 52). Similarly (13), linked problem-focused coping to the educational outcomes of first-year students in Tanzania. Most respondents agreed that they think carefully about the steps to take and concentrate their efforts on addressing the situation they are in, and these factors were shown to improve the mental well-being of university students (48). In the study, problem-focused coping, comprising planning, active coping, or seeking instrumental support, was beneficial for medical students. Problem-focused coping also reduces emotional exhaustion and improves one’s sense of personal accomplishment (27).

There was also moderate agreement that students are actively looking for something good in what is happening, whether it is good or bad. This is a form of positive reinforcement identified in the study by who assessed the influence of cognitive behaviour therapy on students’ stress perceptions in vocational training schools. In the study, cognitive restructuring, reinforcement, motivational enhancement, and mood-monitoring techniques significantly improved mental health outcomes. Other researchers (53) also linked reappraisal and relaxation therapy to improved emotional regulation and resilience among adolescent children in Uganda. Actively engaging with a stressor was also found to improve mood, daily functioning, and students’ quality of life (34).

Another strategy that the students confirmed they employed to deal with stress, albeit moderately, was seeking advice or help from others on what to do about the problem. This form of coping was confirmed to improve people’s mental health (25) and for instance, confirmed that positive framing of a problem that cannot be controlled, and seeking help from more experienced people is instrumental to improving disease management and reducing the incidence of depression among women whose children had been diagnosed with sickle-cell disease (48)conceded that actively seeking instrumental support improves mental resilience of medical students. The current findings also concur with (20), which noted that postgraduate students (peers) may be better positioned to provide the support needed by medical students.

While the analysis results confirmed a negative effect of problem-focused coping on the mental health of post-graduate students, much of the empirical evidence suggests that the right application of problem focused coping such as seeking counsel, advice and encouragement from other people should have positive impacts on an individual’s mental health.

### Emotion-focused coping strategy

The second variable under investigation was on the relationship between the emotion-focused coping strategies on the mental health of postgraduate students at Strathmore University and analysis results revealed a positive relation, with emotion-focused coping having a positive and significant effect on the postgraduate students’ mental health. Neufeld and Malin (2021) (48)considered emotion-focused coping to be an adaptive problem-solving strategy that involves adjusting one’s feelings and emotional response to a stressor and explained that this strategy should improve one’s peace of mind once they accept the state of the stressor’s effects. The finding that emotion-focused coping improves one’s mental health is consistent with the stress and coping theory, which confirms that emotion-focused coping is meant to help change how a stressor is perceived, regulate emotions and alleviate stress (54).

This study’s respondents agreed that they have been learning to live with the problem they are facing, a form of active acceptance that was highlighted in the study by Alanazi et al. (2023) (12) together with other emotion-focused coping strategies such as meditation, therapy, acceptance and seeking social support as among the coping strategies that are more useful when the source of stress is out of the individual’s control. In the study, nurse-led interventions and active emotional coping strategies comprising acceptance, humour, and spiritual practices were shown to reduce anxiety and depressive symptoms among patients diagnosed with heart failure. In China, Wu (2018) (55) also found a positive effect of emotion-oriented coping comprising therapy and acceptance on the mental health of first-time mothers by increasing their motivation to reduce their drug use.

There was also agreement that many of the students are coping by accepting the reality and turning to meditation and prayer for spiritual support. These forms of coping were valuable during the COVID-19 pandemic (14), which revealed that acceptance and other adaptive emotion-based coping strategies had positive impacts on the mental status of students struggling with the anxiety associated COVID-19 restrictions (53) also specified the impact of religious coping and linked it to improved emotional regulation and resilience. Avoidance and emotion-focused coping such as resignation and spiritual focus were linked to an improvement in the mental health of school-going adolescents in Uganda.

While these studies posit that emotion-focused coping improves people’s mental wellness, there is also evidence that they can have adverse effects on an individual. Studies have so far revealed that emotion-oriented coping predicts self-harm among people diagnosed with borderline personality disorder (56). Emotion-focused coping increases the likelihood of aggressive outbursts. The study revealed that people with low self-esteem and cognitive skills are more likely to become violent as they would blame themselves for the problem. Acceptance, prayer, humour, and meditation were identified as positive emotional (57), while wishful thinking, self-blame and venting were confirmed to exert negative effects on a patient struggling with depression. In the current study, a large number of respondents revealed that they were using meditation, acceptance and finding comfort in their religion to cope with stressors linked with increased problem disengagement and had significant negative effects on students’ daily functioning (34).

The finding that it may be better for an individual to accept the gravity of the problem they are experiencing is an important distinction (12). However, findings by Orines and Sunga-Vargas (2023) implied that an increase in perceived stress reduces the positive impact of acceptance, meditation coping and other emotion-based coping strategies (58). Similarly, it has been conceded that a high rate of perceived stress can lead to the use of negative emotion coping such as venting and self-blame, which, in turn, exacerbate mental health conditions (14).

### Avoidant-focused coping strategy

The final coping strategy assessed in this study is the avoidant coping strategy whereby the effects of practices such as self-distraction, denial, substance use, and behavioural disengagement on the mental health of postgraduate students (59, 60). Findings were that these strategies have a positive and significant effect on the mental health of postgraduate students, contradicting the assertions made by the stress and coping theory that this coping style is a maladaptive strategy that should increase stress and anxiety over time. The theory predicts that while avoidant coping behaviours such as procrastination and passive-aggression may provide temporary relief, they do not address the problem and stressor and may exacerbates the stress factor.

From the analysis, student respondents reported that some of the strategies they use to improve their mental wellbeing involve distancing themselves from the stressor by engaging in extracurricular activities such as reading, sleeping, shopping, watching films, playing games, and even swimming. Similar observations indicate that avoiding active confrontation with certain problems could be key to peace of mind (21). The study evaluated the influence of technical avoidance mechanisms that enable people to block online bullies and confirmed that this coping strategy is key to protecting victims. Depending on the frequency of cyberbullying, ignoring the bullies or disconnecting from social media were shown to be effective coping mechanisms that can improve adolescent victims’ mental wellbeing (61). The evidence from these studies confirms that strategic avoidance approaches can indeed lead to a sense of peace and active avoidance of engaging with stressors.

A few respondents further confirmed that they were turning to small projects and work to distract themselves from school stress, which has been linked to calm and a sense of accomplishment (51). Despite this, the researchers went on to confirm that many students deploy harmful practices such as self-criticism, wishful thinking and social withdrawal, which negatively influence their mental wellbeing. They also found that distracting oneself by playing video games is key to relieving pressure, reducing stress and improving one’s acceptance of a stressful event (22).

However, while these studies, and the current find a positive effect of avoidance coping, these findings contradict the observations made by (51, 62, 63) others, whose findings all confirmed that avoidance strategies comprising self-criticism, wishful thinking and social withdrawal all have significant negative effects on stress outcomes. For instance, avoidance strategies such as substance use have a negative effect on students’ motivation and academic performance (62). In the Kenyan university context specifically (64), documented that substance use disorders were significantly associated with major depression, generalised anxiety disorder, and suicidal ideation among first-year university students, underscoring the particular danger of substance-based avoidant coping in populations already carrying elevated psychological risk. These findings are directly comparable to the present study’s cohort, where moderate endorsement of substance use as a coping mechanism (M = 2.94–3.06) warrants targeted institutional attention even when it falls below the majority threshold.

Similarly, linking avoidant coping with depressive symptoms and the onset of problematic drinking. In the study, people who relied on social withdrawal after experiencing a traumatic event experienced increased stress and signs of depressive symptoms (65). The negative effect of this form of social isolation was also reported to exacerbate mental illnesses among university student populations (34) in a study of students accessing a university counselling service, found that avoidant coping strategies — particularly denial, self-blame, and behavioural disengagement — were strongly associated with depression and anxiety, and that self-blame and self-distraction were significant risk factors for suicidal behaviours. This evidence is directly comparable to the present student cohort and underscores why not all avoidant coping is equivalent in its risk profile. From these studies, different types of avoidant coping carry different consequences: leisure-based distraction and temporary disengagement may provide psychological relief, whereas self-blame, denial, and substance use elevate the risk of worsening depression and anxiety among university students. Moreover, it is clear that the effect of avoidant coping depends on the frequency, severity, and degree of control of the patient/victim.

## Conclusions

This study set out to map the mental health landscape of postgraduate students at Strathmore University — a population that is often overlooked in mental health discourse yet carries one of the most complex burden profiles in higher education. At its core, this study documents the mental health consequences of a dual burden: the simultaneous and compounding demands of professional employment, parental and family responsibility, financial self-reliance, and advanced academic rigour. For most students in this cohort, postgraduate study is not a full-time identity but one layer of a multi-layered life — and the psychological cost of sustaining all layers concurrently is both measurable and meaningful. What emerged from the findings is a compelling portrait of students who are not simply studying, but simultaneously working, parenting, self-financing, and striving — and doing so at a psychological cost that is both measurable and meaningful.

The DASS-2 findings made clear that psychological distress among this cohort is neither dramatic nor invisible — it is quiet, persistent, and widespread. Anxiety wears the face of a student dreading public embarrassment in a seminar room. Stress manifests in the inability to switch off after a long day of work and lectures. Depression surfaces in the gradual erosion of enthusiasm, self-worth, and a sense of purpose. These are not clinical emergencies, but they are warning signs — and they are being experienced across the entire student population, not merely at its edges.

The relationship between coping and mental health, confirmed through correlation and regression analyses, demonstrates that how students manage stress matters as much as the stress itself. Emotion-focused and avoidant coping strategies were found to be protective, while over-reliance on problem-focused strategies paradoxically compounded distress — a finding that challenges longstanding assumptions about what constitutes healthy coping in postgraduate education.

Ultimately, this study calls on Strathmore University and comparable institutions across sub-Saharan Africa to listen more carefully to the lived experience of their postgraduate students. The overwhelming majority of students in this study recognised the importance of mental health support — yet most found what was available to them irrelevant to their needs. That gap between demand and fit is where institutional responsibility lies. Building mental health systems that reflect the realities of working students, that destigmatise help-seeking, and that promote adaptive coping as a legitimate academic skill is not a luxury — it is a prerequisite for sustainable postgraduate success. Critically, any institutional response must be calibrated to the dual burden these students carry: generic stress management programmes designed for full-time, financially supported, younger undergraduates will not meet the needs of employed parents pursuing self-financed advanced degrees while managing careers and households. The students are resilient. The question is whether their institutions will rise to meet them.

### Implication for policy and practice

From the study’s conclusions, while postgraduate students experience a lot of stress that contributes to cases of increased mental distress, various effective mechanisms exist. The following recommendations can be drawn from the study’s findings.

The study further recommends that postgraduate students feel comfortable discussing their problems with more experienced faculty and staff members, as these individuals can provide advice that helps alleviate stress. The study also calls on universities to guarantee elements of intervention fit by involving students in the design of these interventions and by ensuring an integrated approach that includes privacy guarantees and the presence of professionally accredited counsellors to psycho-educate students on the main signs of depression and functional coping strategies that students can use to cope with stress in school.

The findings confirm that Emotion-Focused Coping Strategies improve postgraduate students’ mental health. The study recommends educating students on how best to use adaptive emotion-focused coping strategies, such as relaxation techniques, acceptance, and humour, to manage stress. It further recommends that graduate schools promote the use of adaptive emotion-focused coping strategies, including meditation, acceptance, expressing negative feelings, prayer, and learning to live with the stressor, to curb the growing mental health crisis within Kenyan universities.

This study observed that avoidant coping improves the mental health of university students and recommends that students deploy these strategies only when the problem can be avoided or ignored. The study calls on the university to implement training initiatives to inform students about technical strategies to mitigate stressors.

## Data Availability

The raw data supporting the conclusions of this article will be made available by the authors, without undue reservation.
